# Supplementary material for: Characterization of structure and protein of vitelline membranes of precocial (ring-necked pheasant, gray partridge) and superaltricial (cockatiel parrot, domestic pigeon) birds
Source: PLoS One. 2020 Jan 30;15(1):e0228310. doi: 10.1371/journal.pone.0228310 (PMC6992205; doi:10.1371/journal.pone.0228310)
Supplement: Attachment — Submitted filename: PONE-D-19-16659-072419.pdf [file pone.0228310.s004.pdf]

Review article PONE-D-19-16659

In the article entitled « Structure and protein identification of some precocial and superaltricial birds eggs yolk vitelline membrane", the authors explored both the structure and protein profile of vitelline membrane from various birds species. Bird species were selected based on the length of incubation and number of eggs in one clutch (precocial *versus* superaltricial eggs). The authors aimed to correlate the protein and structure specificities of the vitelline membrane to these brooding/hatching characteristics. Although the concept is very interesting, the article deeply lacks convincing/striking conclusions and correlations between experimental observations are either missing or too hazardous. Moreover, the article needs to be edited by an native English speaker (grammatical errors + sentences to be rephrased) and to my opinion, it doesn't fulfill quality requirements for a research article: figure legends are in the main text (at the end of each paragraph), the SEM and MEB pictures are of poor quality and related figures need to be clarified and detailed in related legends. Some data related to chicken egg are missing in Table 1 and in SEM/TEM data considering that you performed proteomic analysis of VM from chicken eggs. The number of proteins identified by mass spectrometry is very low and it is very difficult to perform conclusions as the tables are quite confusing. A Venn diagram comparing results obtained from each species (number of proteins per species, common and specific proteins) would have been clearer.

To conclude, to my opinion, the article is too preliminary to be published in Plos one and requires more integration of the results to be convincing for readers and scientists. There is clearly a lack of data integration and discussion to explain the correlations between the various observations and figures/pictures are of poor quality and are not clear to readers.

Here are some basic comments/suggestions (but not exhaustive) that would be a start to improve the article for a new submission.

Abstract

Please clearly indicate the name of the bird species studied and compared. The number of proteins identified for each species + common and specific proteins have to be mentioned.

Replace "proteomic structure" by "proteomic composition or pattern"

Introduction

Line 48. The vitelline membrane is the matrix for yolk sac expansion over the yolk

Line 51. IL components are expressed by the liver of laying hens but also granulosa cells

Line 62. OCX36 is not specific to the VM but to the eggshell. Apolipoprotein is a yolk-protein. Proteins that seems to be more specifically found in the vitelline membrane are VMO-I and VMO-II (also known as AvBD11)

It would be more convincing to explain the difference in VM structures/composition by phylogenetic analyses rather than on their affiliation to precocial and superaltar birds. Such a hypothesis would be better in the discussion and further prospects.

Please indicate that Gallus gallus, Perdix Perdix and Pahsioanus colchicus are galliforms, Nymphicus hollandicus is a psytaciform and Columba livia a columbiform.

Material and Methods.

Egg collection. Indicate the strain of the laying hens used form proteomics.

Scanning electron microscopy. Please insert a specific paragraph for VM preparation since these preparations were also used for proteomics.

TEM. Indicate what Epon 182 is. Indicate the number of biological replicates

Protein extraction and gel electrophoresis. Line 145. I do not understand the reason for using “15 µL of **enzymatic** proteins”. What is “enzymatic” ?

Results

Eggs morphology:

Table1. use VM thickness instead of width. How did you get this value ? From SEM/TEM data ? Please indicate.

VM Structures

Do not distinguish between precocial and superaltrical birds here. These are results not discussion, please use the respective name of birds (chicken, pheasant, partridge, parrot and domestic pigeon). The use of precocial and superaltrical words may be confusing for non experts.

Line 219. Remove this legend at the end of the article + add details about magnification.

On the figures 1, please indicate A, B, C for each panel and their meaning with respective species to facilitate the reading. . What is the third panel? indicate it in the legend (it appears only in the text).

VM Proteome.

Fig. 5. Remove 30 µL from the legend. This is not informative. The quantity 80 µg at the end of the legend is informative

287. Please start with the number of proteins per species with a Venn diagram showing common and specific proteins between species. Thus you need to perform blast and alignment analyses to identify homologous proteins that may have different protein names depending on the species.

You infer that alpha2-M like 1 is specific to the parrot but at least in chicken you also have an alpha-2 M protein (the last protein of the chicken section in the table) but also in the pheasant (A0A091G4Q7) etc.

Discussion

The discussion has to be rewritten to better show the correlation between structure, physical characteristic and protein composition/abundance.

You may have the same overall protein composition with some subtle difference in sequence but what make the VM structure different may results from proteins interaction that may differ between species and relative abundance.

The presence of “yolk” proteins such as vitellogenins, apolipoproteins” and white proteins (ovalbumin, ovomucin) may rather reflect some yolk/white contamination during VM preparations. Depending on the species, the viscosity of the white may be different and stickier. The conclusions are quite hazardous in this paragraph.

Line 337. VMO-II is written three times.
