# Supplementary material for: Characterization of structure and protein of vitelline membranes of precocial (ring-necked pheasant, gray partridge) and superaltricial (cockatiel parrot, domestic pigeon) birds
Source: PLoS One. 2020 Jan 30;15(1):e0228310. doi: 10.1371/journal.pone.0228310 (PMC6992205; doi:10.1371/journal.pone.0228310)
Supplement: Attachment — Submitted filename: Response to th reviewers.pdf [file pone.0228310.s006.pdf]

Response to the Reviewers

Manuscript: PONE-D-19-16659R1

Dear Editor,

We would like to express our gratitude for assessing our manuscript entitled "Structure and protein identification of some precocial and superaltricial birds eggs yolk vitelline membrane." We conducted the research and prepared the manuscript with the greatest possible precision and care. We have stated the corrections for the specific remarks below. All the amendments have been made to the text and highlighted in yellow. Due to changes in the content of the text, the line numbers may not match those in the unreviewed manuscript.

R: Please change the title to: Structure and protein characterization of the egg yolk vitelline membranes of precocial (common names of the birds???) and superaltricial birds (??)

A: We have changed the title as per the suggestion.

R: L23: "the species": what species?

Also please change the statement to "we analyzed how the structure and protein composition of vitelline membrane (VM) differ among ?? species".

A: We have corrected the statement.

R: L27: please change "enable to" to "be important for counteracting". What do you mean by "complex" some specificity should be given here.

A: We have modified the statement.

R: L29: "triple and three-layer": are these different things or just redundancy?

A: We have removed the words "and three-layer."

R: L30: what is the difference between VM and VM sheets? If not, please use one term consistently throughout the text.

A: We have removed this part of the sentence from the abstract, as it shall not provide any explanation. Sheets are the second structure found following fibers in the OL VM. This has also been discussed in an earlier study of Chung et al. (2010). We have mentioned this in the Results and Discussion (VM structure) sections.

R: L32: are the results for all birds or for one type? Please specify. Please change “weights” to “molecular weights”. What are protein fractions? How are they fractioned?

A: “We found the number of protein fractions to vary from 19 to 23, with molecular weights in the range of 15–250 kDa, depending on the species”—this information pertains to different species of birds.

Protein fractions are individual protein vacuums that are obtained after the SDS-PAGE electrophoresis and have different molecular weights (kDa).

SDS electrophoresis fractionates the proteins according to their mass. This type of electrophoresis is currently the most commonly used and can be applied either individually as an analytical method or as a part of a series of further, more complex studies (e.g. two-dimensional). Fractionation occurs depending on the length of the polypeptide chain, and you can determine the mass of a given protein by comparing with the appropriate standards. This method allows determining the protein mass with an accuracy of 5–10%.

R: L37-39: too much repeats. Please delete them.

A: We have changed the sentence to avoid repetition.

R: L56: Please change the first “is” to “of”.

A: We have corrected the word.

R: L59: please change “so far” to “previously”.

A: We have corrected as per the suggestion.

R: L63: what do you mean by “which are known currently”? Each one of them has been identified?

A: Yes. They are presented in the study of Mann (2008) as “the most comprehensive dataset available at present and complements proteomic analyses of chicken vitelline membrane compartments published previously.”

Mann K. Proteomic analysis of the chicken egg vitelline membrane. Proteomics. 2008; 8: 2322–2332. https://doi.org/10.1002/pmic.200800032 PMID: 18452232.

R: L64: please remove “first” unless you want to say that they have been found elsewhere later and therefore no longer specific to VM.

A: We have removed the word.

R: L67: Please remove “available from studies”

A: We have deleted this text.

R: L70: Please add “the” before “hen”. When referring an animal as a species, please either use “hens” or “the hen”. There are similar mistakes in the rest of the text. Please change them all.

A: All language errors have been corrected by a Native Speaker English (Translmed Publishing Group).

R: L72: what is the course of the fibers? Do you mean pattern?

A: We have substituted the word “course” by “pattern.”

R: L73: “Additional structures” such as ???

A: These structures do not have a name. They appear in the form of tabs, but this is not an official term. In the cited literature, they are depicted in the SEM micrographs.

R: L76: “offspring ones”? do you mean “offspring”?

A: We have deleted the word “ones.”

R: L77: “the authors”: are you still referring those that published the studies you mentioned earlier? From the context, “the authors” in L71 referred to them.

A: We have corrected the sentence.

R: L80: Please change “The birds” to “Birds”. Please also add an explaining phrase after “precocial” (just like you did for the other three categories) to be parallel and coordinated.

A: We have provided an explanation in the sentence.

R: L83: please change “first days” to “first few days”.

A: We have corrected as per the suggestion.

R: L85: please change “the chicks” to “chicks”.

A: We have corrected as per the suggestion.

R: L88: using % for egg yolk and water does not make sense. Because water is part of the yolk. At least change the “a large proportion” to “a high percentage” so people are not misled into thinking you are talking about the two proportions of the eggs.

A: We have removed the information on water content in eggs.

R: L93: “a few “ and “several” are both unspecified and don’t differ much. I suggest you just use one of them.

A: We have corrected the sentence.

R: L94: please change “storage” to “delayed incubation”. By “storage” you gave the impression that the eggs were taken and stored deliberately.

A: We have changed the word.

R: L98: Please add “using ?? and ?? as models” before “We”.

A: We have changed the sentence as follows:

“We also identified the proteins present in VM using the NanoAcquity Ultra Performance LC (Waters) system.”

R: L112: Please remove “to be” and add “because fertile males were present” (if this is the case).

A: We have changed the text.

R: L181: molecular weights? Please change all such occurrences in the following sections.

A: We have corrected as per the suggestion.

R: L183: please remove “used”.

A: We have removed the word.

R: At the ends of the sections for SEM, TEM and Protein identification, please add a statement of the number of samples used. For example, “Ten eggs from each of the 4 species were analyzed yielding a total samples of 40”.

A: We have corrected the data as follows:

“Six eggs from each of the 4 species were analyzed yielding a total sample of 24.”

R: L190: please change to “Egg and VM characteristics”

A: We have changed the subtitle.

R: L191: Please change “morphological traits” to “weight characteristics”. There were no morphology characterization.

A: We have changed the statement.

R: Table 1: please change “Results of the comparative analysis of the morphological” to “Weights”.

A: We have changed as per the suggestion.

R: Fig 3: From the labeling it appears that CM is a very thin layer of membrane with little characteristics to be seen from the figure. What did the authors use to identify this layer? Please describe its features and characteristics.

A: We agree that CM is poorly visible, and therefore, we did not determine it in the SEM images in the first version of our manuscript. However, one reviewer suggested that CM can be determined, as has been done in earlier studies. CM forms a very thin membrane between the IL and OL. Using an example, we would like to show the reviewer how CM has been determined in other studies, which were used as a model for ours. The below scan presents the CM determined in the publication of: Kido S, Doi Y. Separation and properties of the inner and outher layers of the vitelline membrane of hen’s eggs. Poult Sci. 1988; 67: 476–486. https://doi.org/10.3382/ps.0670476.

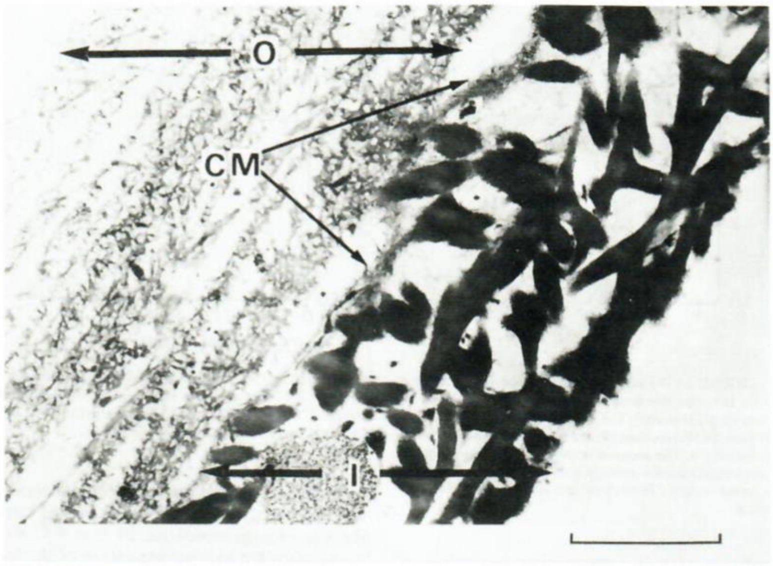

FIGURE 3. Transmission electron micrograph of cross-section of the vitelline membrane from a fresh egg. I; inner ayer, 0; outer layer, CM; continuous membrane. Bar indicates 2 μ.

R: L246-247: “All the three layers were also characterized by a layered structure”. This statement does not add anything unless the authors did not state it clearly. They have already described in previous statements that the VM were triple-layered.

A: We have deleted the sentence.

R: In the text, “Fig” was used but in the figure legend, “Fig.” was used, please be consistent and I suggest Fig. be used because it is abbreviated.

A: We have changed “Fig” to “Fig.” consistently in the manuscript.

R: Fig 4 was not provided in the complied file.

A: We have provided Fig. 4.

R: L254: please change “corresponded to” to “formed”

A: We have changed the word.

R: L278: Please change “in” to “for”, please also remove “of proteins”

A: We have changed as per the suggestion.

R: L298-300: Fig 5 legend. Please explain what the red arrows are.

A: We have explained this in the caption of Fig. 5.

R: L305-317: please move this section to after Table 3

A: We have moved the entire text below Table 3.

R: L318: what bands were selected? Are those the red arrowed ones? Please specify.

A: Yes. We have provided this information in the text.

R: L319: please remove “in the study”

A: We have removed the text.

R: L322 and 324: 4 proteins >250 kDa and 3 proteins of 35kDa were found. Later only one of each were described. Please explain the others.

A: Proteomic analysis confirmed that the 250-kDa protein found in the VM of all the birds analyzed was alpha-2-macroglobulin-like 1 protein, which is an endopeptidase inhibitor or

mucin 5B. In the case of partridge and pheasant, this protein was observed at the lowest intensity.

R: L325: please add "ZP3" here

A: We have completed the sentence.

R: L310-311: In Fig 5, proteins of the complement system were not noted. Which ones are referred to here?

A: In Fig. 5, we present the entire protein profile of the VM of individual cacti (supplementary materials). For further detailed proteomic analysis, we selected the individual numbered protein fractions.

R: L333: similarly, 3 proteins were observed but only one was described. What happened to the other two?

A: In the case of gray partridge and cockatiel parrot, only three and seven proteins were observed, respectively (Fig. 7). The 15-kDa protein band obtained after the separation of the VM of domestic pigeon demonstrated the presence of H0Z0C5 protein, the function of which has not yet been identified.

The data are presented in the supplementary materials and the Venn diagram.

R: Tables 2 and 3: please change pH, PLGS score, and % of coverage to contain one decimal point. Please change the molecular weight to kDa to be consistent with the text. Also in both tables for most protein entries, portions of the IDs were presented twice consecutively. For example: A0A2P4SB66 A0A2P4SB66. What is the reason for this annotation?

A: We have corrected everything and removed the IDs that were repeated by mistake.

R: Table 3: please indicate which section corresponds to which red arrow. If all VM proteins were identified and presented in Table 2 (as indicated in the title of Table 2), why list them again in Table 3? Are these presented for a second time? If so, there is no need to have this Table.

A: The red arrows represent the protein fractions that were selected for the detailed proteomic analysis of the VM birds. The detailed analysis of these fractions is presented in the supplementary material S3. We believe that all the results should remain in the article.

R: "after ovulation in the ovarian follicle" in Line 373 in the corrected manuscript should be corrected to "after ovulation in the infundibulum".

A: We have changed the text.
