# Supplementary material for: Characterization of structure and protein of vitelline membranes of precocial (ring-necked pheasant, gray partridge) and superaltricial (cockatiel parrot, domestic pigeon) birds
Source: PLoS One. 2020 Jan 30;15(1):e0228310. doi: 10.1371/journal.pone.0228310 (PMC6992205; doi:10.1371/journal.pone.0228310)
Supplement: Attachment — Submitted filename: Response to th review.docx [file pone.0228310.s005.docx]

Response to the Review

Manuscript: PONE-D-19-16659

Dear Editor

We would like to express our gratitude for the assessment of our manuscript entitled „Structure and protein identification of some precocial and superaltricial birds eggs yolk vitelline membrane”. Conducting of the research and preparing of the publication were made by us with the greatest possible precision and care. We would like to point out that for the authors: Krzysztof Damaziak and Marek Kieliszek the affiliations have been changed. This is due to the name changes of our University Faculties from October 1, 2019. Below are corrections for the specific remarks. All the amendments were made to the text and marked with a yellow background. Due to changes in the content of the text, line numbers may not match those before the review:

Reviewer: 1

R: lines 19-38: Syntactic complexity or errors throughout in "ABSTRACT" section should be improved.

A: English in the entire manuscript has been re-verified and corrected by a native speaker. We have attached a suitable certificate in the supplemental materials.

R: Lines 50-52 and 56-57: There might be some inconsistencies in "Introduction" section. I guess the IL is mainly composed of zona pellucida glycoproteins but not collagen.

A: It has been corrected

R: Lines 320 and 322: CM should be mentioned and explained in the "Introduction" section and be indicated in the Figure 3.

A: In the chapter Introduction we have added the following sentence ‘Between them lies a granular “continuous membrane” (CM – lamina continua) of unreported composition [1].’ We have marked CM in Figure 3 and introduced an explanation in the figure caption.

R: Lines 331-335: In chicken and Japanese quail both being included in the precocial birds, either IL or OL appears to be the single layer. According to the authors hypothesis, they should have multiple layers. How to explain the previous observations in chicken and quail VM? Is it possible to explain that the VM of precocial birds are folded after OL formation?

A: Unfortunately, at the moment we are unable to precisely explain the observations we obtained. The image of three-layer VM of pheasant and partridge was a great surprise to us. We repeated the analyses several times and the TEM image was always similar. It is surprising because in the case of chicken and partridge VM, the structure is indeed single, as pointed out by the reviewer. What is more, we are currently conducting VM analyses of other avian species, including Turdus merula, Turdus philomelos, Rhea americana, Cairina moschata and despite the considerable morphological diversity, VM only cosnsists of single layers. We suspect that this can be related to the evolutionary adaptation of nesting strategies (e.g. high number of eggs per clutch). As we have attempted to explain this in the discussion, the nesting behavior of pheasant and partridge differ from those observed in, among others, wild ancestors of the domestic hen. We suspect, that the answer may be brought by histological tests of the oviduct and VM with the use of various staining types. We will seek to clarify these observations in future studies.

R: Lines 493-506: It might be better that "Conclusion" section contains the conclusion bringing together the results from all the experiments in this study not only from the proteomic ones.

A: We have completed the „Conclusion” as suggested by the reviewer

R: Line 59: "apovitellin" should be replaced with "lipovitellin".

A: We replaced "apovitellin" to "lipovitellin"

R: Lines 259-277: "protein fraction" might be replaced with "protein bands" or "protein signals".

A: It has been corrected

R: Line 262: "first line" should be replaced with "first lane".

A: We replaced "line" to "lane”

R: Lines 262-263: "egg whites VM" might be replaced with "egg yolks VM".

A: We replaced "whites" to "yolks”

R: Lines 328, 329, 334 and 335: "oocyte infundibulum" or "infundibulum" should be replaced with "ovarian follicle" or "follicle". "infundibulum" is a part of oviduct.

A: It has been corrected

R: Line 328: Is "they" mean ZP1? If so, "they" should be replaced for eample with "one of them".

A: In the indicated fragment we have introduced the following sentence: “According to Bausek et al. [29] at least one of the major IL components - chkZP1 is synthesized in the liver and is transported via the bloodstream to the ovarian follicle.”

R: Line 484: "zona pellucida binding proteins" might be replaced with "zona pellucida sperm binding proteins".

A: It has been replaced

R: Lines 494 to 495: Bird species names of the precocial and altrical birds might be exchanged.

A: It has been corrected

Reviewer: 2

R: The authors aimed to correlate the protein and structure specificities of the vitelline membrane to these brooding/hatching characteristics. Although the concept is very interesting, the article deeply lacks convincing/striking conclusions and correlations between experimental observations are either missing or too hazardous.

A: We agree with the reviewer that the conclusions and discussion may be dangerous. However, this is the first report describing possible differences resulting from the structure and protein content in the VM of individual bird species. We've improved results discussions to provide new information. At the same time, we described the Venn diagram that will make the description of the results more attractive.

R: Moreover, the article needs to be edited by an native English speaker (grammatical errors + sentences to be rephrased) and to my opinion, it doesn’t fulfill quality requirements for a research article: figure legends are in the main text (at the end of each paragraph)

A: The article was corrected by Native English Speaker (www.translmed.com). Figures and legends have been included in the text in accordance with the requirements of the magazine PlosONE.

R: The SEM and MEB pictures are of poor quality and related figures need to be clarified and detailed in related legends. Some data related to chicken egg are missing in Table 1 and in SEM/TEM data considering that you performed proteomic analysis of VM from chicken eggs. The number of proteins identified by mass spectrometry is very low and it is very difficult to perform conclusions as the tables are quite confusing. A Venn diagram comparing results obtained from each species (number of proteins per species, common and specific proteins) would have been clearer.

A: TEM and SEM images were prepared in line with the PLOS ONE journal guidelines. We perceive them as a very high quality - clear and sharp. Their quality is deteriorated after sending them to the journal and PDF generation. Unfortunately, this is beyond our control. In line with the reviewer’s suggestion, we have developed two Venn diagrams: Figure 6 in which we have presented the amount of proteins defined for individual species and the common proteins. Figure 7 presenting specific proteins defined based on the selected bands. This study did not analyze the VM structure of chicken egg yolk with the use of SEM and TEM. This stems from the fact that such studies have been conducted and published on numerous occasions. Therefore, we assumed that this kind of information is well known and one can use the available knowledge with full confidence. Restriction of the analysis by excluding the hen VM structure analysis, which is expensive, labor-intensive and time-consuming, enabled us to better focus on VM analyses of the remaining 4 species. This was highly important for us, as none of the described analyses has ever been performed for either ring-necked pheasant, grey partridge, cockatiel parrot, or domestic pigeon. However, we decided to perform protein analyses for hen VM as such data have been published only on several occasions, and we were aiming at a comparison. In the study of protein identification, we have treated hen VM as reference and bridge between our results and the literature knowledge.

R: Figures/pictures are of poor quality and are not clear to readers.

A: All Figures/pictures have been prepared in line with the PLoS ONE Author Guidelines. Their deteriorated quality stems from processing and PDF file development in the journal's system. This is beyond author's control. The minimum photo resolution is 300x300 DPI.

R: Abstract: Please clearly indicate the name of the bird species studied and compared. The number of proteins identified for each species + common and specific proteins have to be mentioned. Replace “proteomic structure” by “proteomic composition or pattern”

A: We have added full avian species names in the abstract. We have provided the amount of proteins identified per species. We have not mentioned all proteins common between individual species because this prohibited by the editorial limitation of the word count for this chapter. This has been described in detail in the results chapter based on the attached Venn diagrams. We have replaced “proteomic structure” with “proteomic composition or pattern”. However, we suggest a complete deletion of this sentence. In our opinion it is not necessary in the abstract and we may not exceed the number of 300 words.

R: Introduction: Line 48. The vitelline membrane is the matrix for yolk sac expansion over the yolk.

A: It has been corrected

R: Introduction: Line 51. IL components are expressed by the liver of laying hens but also granulosa cells

A: The text has been completed

R: Introduction: Line 62. OCX36 is not specific to the VM but to the eggshell. Apolipoprotein is a yolk-protein. Proteins that seems to be more specifically found in the vitelline membrane are VMO-I and VMO-II (also known as AvBD11)

A: A: Of course, apolipoprotein is a yolk protein but it has also been identified in the hen's egg membrane (Mann, 2008). It should be noted that depending on the species of bird, the content of this protein (apolipoprotein) may be different in the vitelline membrane.

We agree with the reviewer. Of course (VMO-I and VMO-I) these are the most popular proteins found in vitelline membrane.

Research presented by Gautron (2011) showed that the OCX36 protein is eggshell-specific protein that is secreted by the regions of the oviduct responsible for eggshell formation. It has also been identified in vitelline mebrane.

Mann, K. (2008). Proteomic analysis of the chicken egg vitelline membrane. Proteomics, 8(11), 2322-2332.

Gautron, J., Rehault-Godbert, S., Pascal, G., Nys, Y. & Hincke, M. T. (2011). Ovocalyxin-36 and other LBP/BPI/PLUNC-like proteins as molecular actors of the mechanisms of the avian egg natural defenses. Biochemical Society Transactions, 39(4), 971-976, doi:10.1042/BST0390971

R: Introduction: It would be more convincing to explain the difference in VM structures/composition by phylogenetic analyses rather than on their affiliation to precocial and superaltar birds. Such a hypothesis would be better in the discussion and further prospects.

A: Our aim was to demonstrate differences depending on the nesting specificity of birds. Analysis of structure and composition of VM through phylogenetic analysis would be indeed highly interesting and we will seek to perform it in the future. However, it seems that it is too early for such an analysis. The available literature lacks information on VM of other birds than chicken, partridge and duck. Our team has recently expanded the available knowledge on the analysis of VM ratite birds (ostrich, emu and rhea). Now we have selected 4 further species for which the VM analyses are performed for the first time. We are currently analyzing the structure and protein composition of VM of Turdidae species. We hope that soon we will be able to conduct a phylogenetic meta-analysis. In the Material and methods chapter we have supplemented the information on the classification of birds to given families, but we would like to keep the main objective of the study without major changes.

R: Introduction: Please indicate that Gallus gallus, Perdix Perdix and Pahsioanus colchicus are galliforms, Nymphicus hollandicus is a psytaciform and Columba livia a columbiform.

A: We have supplemented the information. We have introduced names of systematic orders but we believe that this will be more appropriate in the M&M chapter. Of course, we agree with the reviewer this is the next stage (with more different species of birds) of the research we carry out. In this work we have added a special chart: Venn diagram - a diagram to illustrate the relationship between sets.

R: Material and methods: Egg collection. Indicate the strain of the laying hens used form proteomics.

A: Data has been corrected. Information about the hen strain (ISA BROWN) from which eggs were used analyzes was supplemented.

R: Material and methods: Scanning electron microscopy. Please insert a specific paragraph for VM preparation since these preparations were also used for proteomics.

A: We have inserted a separate chapter on VM preparation.

VM samples for SEM analysis were prepared following the methodology described by Kirund and McKee [20]. After breaking the shell, the egg content was poured onto a separator to separate the egg yolk from the white. After weight determination (± 0.1 g), yolk was placed on a glass pan so that the germ disc was visible on the surface. VM was cut with a scalpel around the egg yolk about halfway up. The VM was then rinsed in deionized water (~4°C) until all residues of the yolk visible to the naked eye were removed The weight of the whole VM (±0.1 mg) was determined and then the area of the embryonic disc (not analyzed area) was separated.

Protein identification is presented in a separate chapter: Protein identification

R: Material and methods: TEM. Indicate what Epon 182 is. Indicate the number of biological replicates

A: Eight biological replicates were performer.

R: Material and methods: Protein extraction and gel electrophoresis. Line 145. I do not understand the reason for using “15 μL of enzymatic proteins”. What is “enzymatic” ?

A: This is a mistake in translation. Of course, there should be only "proteins".

R: Results: Eggs morphology: Table1. use VM thickness instead of width. How did you get this value ? From SEM/TEM data ? Please indicate.

A: The VM thickness was measured on TEM images via Nis Elements version 5.10, Nikon optical microscope (type 104c, Japan).

R: Results: VM Structures. Do not distinguish between precocial and superaltrical birds here. These are results not discussion, please use the respective name of birds (chicken, pheasant, partridge, parrot and domestic pigeon). The use of precocial and superaltrical words may be confusing for non experts.

A: This has been corrected

R: Results: Line 219. Remove this legend at the end of the article + add details about magnification.

A: Figure legends are introduced in the text in place for the proposed presentation. They have been introduced in this manner because such are the requirements of the PLoS ONE Submission Guidelines.

*„Figure captions*

*Figure captions must be inserted in the text of the manuscript, immediately following the paragraph in which the figure is first cited (read order). Do not include captions as part of the figure files themselves or submit them in a separate document.”*

All magnifications SEM and TEM are present in the photographs with automatic caption. We understand that they were not visible due to the quality change of these files. Properly formatted micrograms are supplied to the editorial office, thus we believe that if the manuscript is published, they will be clearly visible.

R: Results: On the figures 1, please indicate A, B, C for each panel and their meaning with respective species to facilitate the reading. . What is the third panel? indicate it in the legend (it appears only in the text).

A: Photos must have been formatted incorrectly when building the PDF. In each photo, information is added: precocial or superaltrical. All photos have a resolution of 300x300 DPI.

R: Results: VM Proteome. Fig. 5. Remove 30 μL from the legend. This is not informative. The quantity 80 μg at the end of the legend is informative

A: It has been removed

R: Results: 287. Please start with the number of proteins per species with a Venn diagram showing common and specific proteins between species. Thus you need to perform blast and alignment analyses to identify homologous proteins that may have different protein names depending on the species.

A: This has been corrected. We have changed the discussion of the results obtained. We also made a Venn diagram.

The conducted characteristics of the proteins in the vitelline membrane with the use of Venn diagram (Fig. 6) for hen demonstrated 14 proteins, which have not been determined in the VM of all birds. In the case of pheasant VM 7 proteins were identified, which were absent from other birds. It should be emphasized that the protein structure of pheasant VM was most closely related in terms of the presence of proteins present in the VM of other birds as well. The most pronounced similarity of pheasant VM was determined for grey partridge (5 proteins) and chicken (2 proteins). The lowest number of proteins (6) among the analyzed avian VM was found in cockatiel parrot. It should be emphasized, that the presence of the same protein (A0A2IOTKM1) was found for cockatiel parrot, ring-necked pheasant and pigeon. Domestic pigeon VM analysis demonstrated the highest number of protein bands (23).

In addition, individual protein bands obtained through electrophoretic separation (Fig. 5) were selected in the study and were subjected to an in-depth analysis in terms of the presence of specific proteins (Fig. 7). The conducted analysis demonstrated the highest number of 4 proteins in the case of the protein band (>250 kDa) of VM isolated from cockatiel parrot egg and 3 proteins from the protein band with the weight of approx. 35 kDa.

R: Results: You infer that alpha2-M like 1 is specific to the parrot but at least in chicken you also have an alpha-2 M protein (the last protein of the chicken section in the table) but also in the pheasant (A0A091G4Q7) etc.

A: Thank you to the reviewer for the information. The data has been corrected. We have deleted invalid data. This protein was also confirmed in an additional analysis. Supplementary S3 Data file.

R: Discussion: The discussion has to be rewritten to better show the correlation between structure, physical characteristic and protein composition/abundance.

A: A: In the discussion, chapters about proteomics and physical features were written in separate chapters. After all, we've added new information. We believe that the combination of all this information could affect the occurrence of chaos and inconsistencies while reading the article. We have determined that such a division will be the most appropriate.

The abundance character of individual proteins obtained from avian VMs demonstrated association with the structure and individual physical traits. In the case of pigeon, for which the highest number of proteins was obtained, we determined that their abundance could be decisive for its different physical parameters. The identified single common proteins that have also been found in parrot, pheasant and partridge did not have such a pronounced impact on the similar structure towards the tested VM membranes. In the case of the structure of parrot VM, it also differed considerably in morphological terms from other VMs isolated from bird eggs. This can be confirmed by the presence of 6 proteins, which were absent from other birds. The similar structure of partridge and pheasant VM should be emphasized, which may indicate the presence of 5 common proteins. However, it should be emphasized that an in-depth analysis of proteomic and physical linkages between individual avian membranes should be continued to obtain the highest possible amount of information on the differences occurring between individual bird species. Such knowledge will enable us to understand and reveal numerous differences. Furthermore, it will allow noticing individual traits present between bird species, which can be of high significance from the proteome standpoint. Obtaining such information may be the basis for the definition of their functions and properties.

R: Discussion: You may have the same overall protein composition with some subtle difference in sequence but what make the VM structure different may results from proteins interaction that may differ between species and relative abundance.

A: Of course, we agree with the reviewer's suggestion. Protein interactions occurring in the VM membrane can change its structural structure. Determination of individual protein sequences and their conformations in the PyMol program and their impact on the VM structure - this is the topic for the next scientific article that we will prepare in the future. We want to perform an in-depth analysis of individual VM membranes. Many proteins have not yet been fully characterized and their function is still unknown. This is a new topic that will allow you to deepen knowledge about the construction of the VM and its role.

R: Discussion: The presence of “yolk” proteins such as vitellogenins, apolipoproteins” and white proteins (ovalbumin, ovomucin) may rather reflect some yolk/white contamination during VM preparations. Depending on the species, the viscosity of the white may be different and stickier. The conclusions are quite hazardous in this paragraph.

A: We agree with the reviewer that the conclusions may be dangerous. However, we think this is the first report on the analysis of proteins isolated from the VM of various birds. Vitellin membrane has been very carefully prepared. Several membrane extractions were performed.

R: Line 337. VMO-II is written three times.

A: It has been corrected
