# Supplementary material for: Characterization of structure and protein of vitelline membranes of precocial (ring-necked pheasant, gray partridge) and superaltricial (cockatiel parrot, domestic pigeon) birds
Source: PLoS One. 2020 Jan 30;15(1):e0228310. doi: 10.1371/journal.pone.0228310 (PMC6992205; doi:10.1371/journal.pone.0228310)
Supplement: Attachment — Submitted filename: Response to th reviewers.DOCX [file pone.0228310.s007.DOCX]

Response to the Reviewers

Manuscript: PONE-D-19-16659R2

Dear Editor,

We would like to express our gratitude for assessing our manuscript entitled “Characterization of structure and protein of vitelline membranes of precocial (ring-necked pheasant, gray partridge) and superaltricial (cockatiel parrot, domestic pigeon) birds.” We conducted the research and prepared the manuscript with the greatest possible precision and care. We have stated the corrections for the specific remarks below. All the amendments have been made to the text and highlighted in yellow. Due to changes in the content of the text, the line numbers may not match those in the unreviewed manuscript.

R: I strongly recommend the authors to polish the draft more and more. For example, there are quite similar contents, "... is primarily composed of glycoproteins of the zona pellucida", "The components of IL (mainly glycoproteins of the zona pellucida)" and "The IL primarily consists of glycoproteins, five of which have been identified..." in lines 52, 55 and 60, respectively. For other example, the order of bird species in lines 39 to 40 do not depend on that in lines 36 to 37. The authors should arrange context to reduce such unnecessary repeats and disorders to make the arguments clear.

A: We have carefully checked the entire manuscript. All similar contents has been removed. The names of the birds have also been standardized and are now presented in the same order wherever possible.
